# Maps of cropping patterns in China during 2015–2021

**DOI:** 10.1038/s41597-022-01589-8

**Published:** 2022-08-05

**Authors:** Bingwen Qiu, Xiang Hu, Chongcheng Chen, Zhenghong Tang, Peng Yang, Xiaolin Zhu, Chao Yan, Zeyu Jian

**Affiliations:** 1 Key Laboratory of Spatial Data Mining &Information Sharing of Ministry of Education, Academy of Digital China (Fujian), Fuzhou University, Fuzhou, 350116 Fujian China; 2 Community and Regional Planning Program, University of Nebraska-Lincoln, Lincoln, 68558 Nebraska USA; 3 Key Laboratory of Agricultural Remote Sensing, Ministry of Agriculture and Rural Affairs, Beijing, China; 4 Department of Land Surveying and Geo-Informatics, The Hong Kong Polytechnic University, Hong Kong, China

**Keywords:** Agriculture, Biogeography

## Abstract

Multiple cropping is a widespread approach for intensifying crop production through rotations of diverse crops. Maps of cropping intensity with crop descriptions are important for supporting sustainable agricultural management. As the most populated country, China ranked first in global cereal production and the percentages of multiple-cropped land are twice of the global average. However, there are no reliable updated national-scale maps of cropping patterns in China. Here we present the first recent annual 500-m MODIS-based national maps of multiple cropping systems in China using phenology-based mapping algorithms with pixel purity-based thresholds, which provide information on cropping intensity with descriptions of three staple crops (maize, paddy rice, and wheat). The produced cropping patterns maps achieved an overall accuracy of 89% based on ground truth data, and a good agreement with the statistical data (R^2^ ≥ 0.89). The China Cropping Pattern maps (ChinaCP) are available for public download online. Cropping patterns maps in China and other countries with finer resolutions can be produced based on Sentinel-2 Multispectral Instrument (MSI) images using the shared code.

## Abstract (summary)

**Table:** 

Measurement(s)	area of different cropping patterns
Technology Type(s)	phenology-based approach
Factor Type(s)	type of cropping pattern
Sample Characteristic - Organism	Organism
Sample Characteristic - Environment	cultivated environment
Sample Characteristic - Location	China

## Background & Summary

Global food security is the most important issue in human society, especially in the most populated country, China^1^. Agricultural intensification through multiple cropping with diverse crop species can significantly increase crop production as well as reduce the associated environmental consequences^2,3^. Around 12% of global croplands experience multiple cropping and among them, 34% of rice lands are under multiple cropping systems^2^. China feeds about 20% of the world’s population with only 7% of the world’s farmland^1^. Around one-third of croplands are cultivated by multiple crops in China^4,5^, which is twice of the global average^2^. China ranked first in global cereal production^6^. Paddy rice, maize, and wheat are the most important staple cereal crops in China. On a global scale, these three staple crops accounted for 79% of the total harvested cereal areas^6^. In China, the three staple crops contributed to around 97% of national cereal areas in 2020 (www.stats.gov.cn/english/). Developing a spatially explicit multiple cropping dataset with information on crop types is important for ensuring agricultural sustainability^2^.

Remote sensing has long been applied to produce maps of cropping intensity and crop types in recent decades^7,8^. However, national-scale agricultural mapping remains challenging due to a lack of sufficient training samples required for mapping algorithms continuously over large areas^9^. The phenology-based approaches have been developed for crop mapping by analyzing the crop life cycle, relieving the reliance on training samples^10^. Phenology-based algorithms were commonly developed based on the temporal profiles of Vegetation Indices (VI), which reflected unique phenology for each specific crop^11,12^. Nevertheless, the phenology-based algorithms over large spatial domains and multiple years needs to deliver the challenges of intra-class variability and inter-class similarity in spectral and temporal characteristics^13^. Several strategies have been proposed to cope with these challenges, such as automatically detecting the key phenological stages and unique cropping practices through incorporating multiple spectral indices and multi-resources data^14,15^. MODIS images have long been successfully exploited in agricultural applications due to the advantages of temporal resolutions^11,16^. However, the mixed-pixel problem of MODIS images needs to be addressed^17^. The precise agricultural mapping is constrained by the lack of images with fine-resolution and adequate revisit frequency^9^. There is a need to expand the cropping intensity mapping strategy through the incorporation of crop description^2^.

Cropping systems have changed significantly in response to agricultural policies, global food prices, and climatic changes^18^. The Chinese government has implemented a series of strong policy measures to stimulate agricultural production, such as agricultural subsidizing and price support targeted to major food crops^19^. As a result, China has experienced dramatic changes in agricultural production structure in recent decades^20^. The historical high maize stock in 2015 has become one of the biggest agricultural problems for recent years^19^. Therefore, the Chinese government accelerated agricultural supply-side reform to optimize the agricultural structure^1^. However, there is a lack of useful references on how the agricultural supply-side reform influenced agriculture structure^19^. There is a lack of reliable updated nation-scale maps with detailed descriptions on cropping intensity and crops rotations in China in recent year^21^. This study aimed to fill this scientific data gap by providing annual national maps on Cropping patterns in China during 2015–2021 based on MODIS images using phenology-based mapping algorithms. Pixel purity-based thresholds were proposed and applied in the decision rules in order to cope with the mixed pixel problem of MODIS images.

## Methods

### Study area

**Fig. 1:**
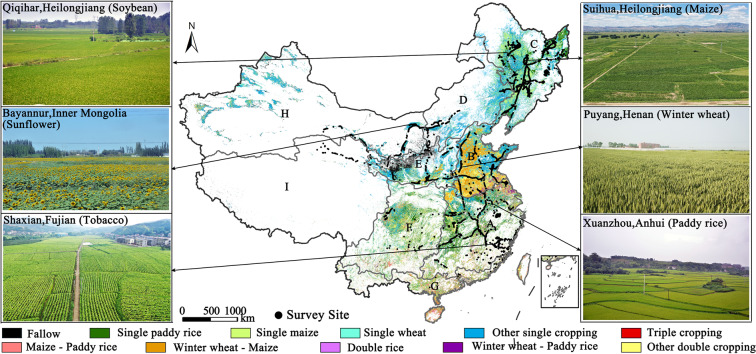
The distribution map of cropping patterns in 2021, 9 agricultural regions and validation sites in China. Notes: A to I represented nine agricultural regions in China. (**A**) Middle-lower Yangtze River Plain; (**B**) Huang-Huai-Hai plain; (**C**) Northeast China; (**D**) Inner Mongolia and along the Great Wall; (**E**) Loess plateau; (**F**) Southwest China; (**G**) Southern China; (**H**) Gansu-Xinjiang region; (**I**) Qinghai-Tibet region.

There is a long history of diversified cropping patterns due to the climatic and topographic complexity in China^4^. Cropping intensity increases from north to south, and multiple cropping dominates in regions south of 40^0^N^4^. For example, multiple cropping systems of double rice and winter wheat plus maize are popular in the Middle-lower Yangtze river plain and the Huang-Huai-Hai plain, respectively (Fig. 1)^22^. Three staple crops, maize, paddy rice, and wheat, are widely distributed across the country (Figure S1). These three major crops contributed to more than half (57.08%) of the total sown area in China in 2020 (http://www.stats.gov.cn/english/).

### MODIS images and pre-processing

**Fig. 2:**
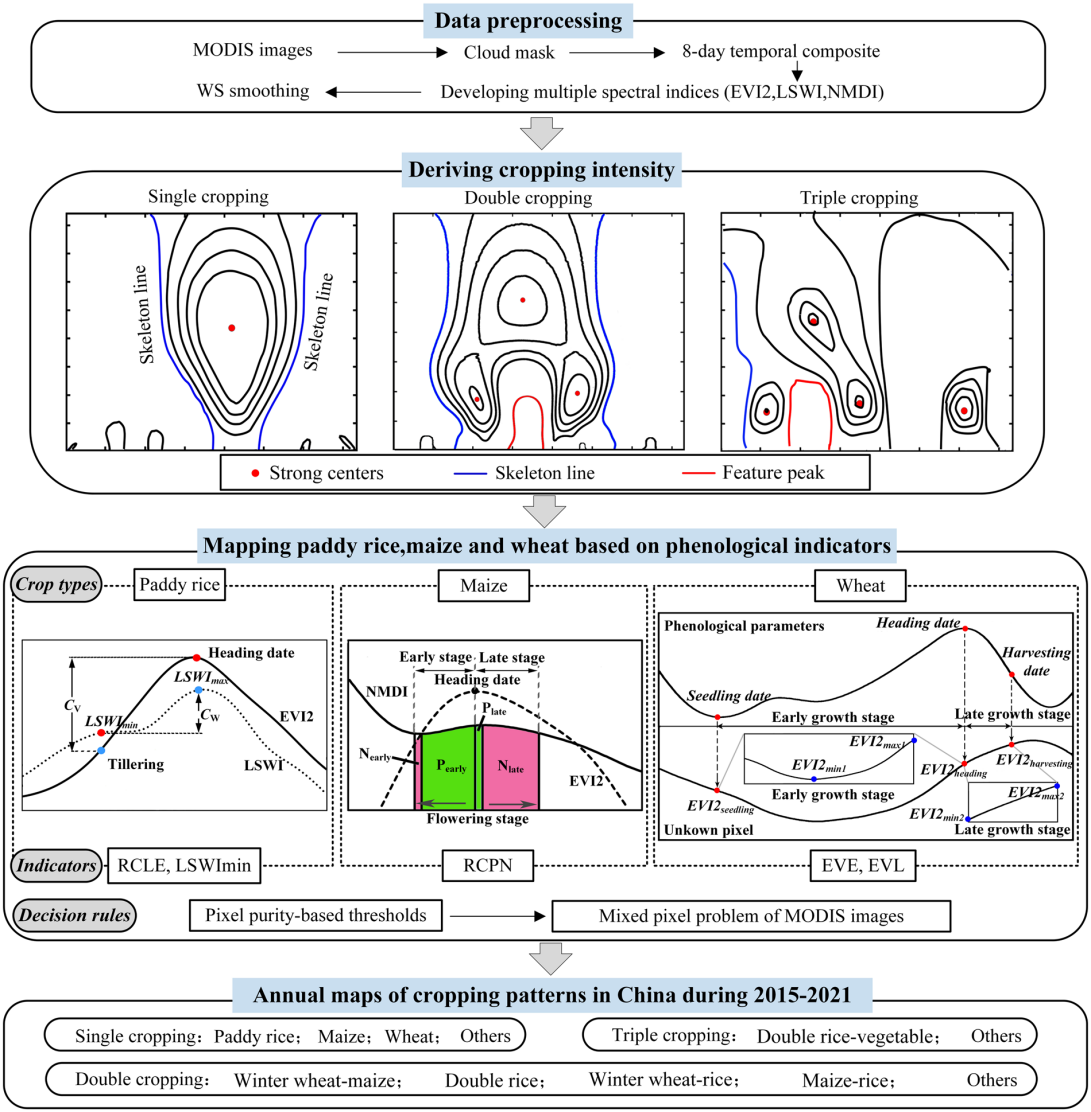
The workflow of the methodology: Data preprocessing, deriving cropping intensity, mapping three staple crops and obtaining annual maps of cropping patterns in China.

We used the 500 m 8-day composite Moderate Resolution Imaging Spectroradiometer (MODIS) surface reflectance products (MOD09A1) from 2015 to 2021. Three spectral indices were calculated: the 2-band Enhanced Vegetation Index (EVI2)^23^, LSWI^16^, and Normalized Multi-band Drought Index (NMDI)^24^ (Fig. 2). The functions of EVI2, LSWI, and NMDI are provided in Eqs. 1–3 as follows.

1 $${\rm{EVI2}}=2.5\times \left({\rho }_{NIR}-{\rho }_{{\rm{Red}}}\right)/\left({\rho }_{NIR}+2.4\times {\rho }_{{\rm{Red}}}+1\right)$$

2 $${\rm{LSWI}}=\left({\rho }_{NIR}-{\rho }_{SWIR6}\right)/\left({\rho }_{NIR}+{\rho }_{SWIR6}\right)$$

3 $$NMDI=\frac{{\rho }_{NIR}-\left({\rho }_{SWIR6}-{\rho }_{SWIR7}\right)}{{\rho }_{NIR}+\left({\rho }_{SWIR6}-{\rho }_{SWIR7}\right)}$$

where, *ρ*_*NIR*_, *ρ*_Red_, *ρ*_*SWIR6*_ and *ρ*_*SWIR7*_ represented the surface reflectance values from the red (620–670 nm), Near-infrared (841–875 nm), short wave infrared band centered at 1640 nm (1628–1652 nm) and 2130 nm (2105–2155 nm), respectively.

For each spectral index (EVI2, LSWI, and NMDI), a daily continuous time series was developed based on the cloud-free observations using the Whittaker Smoother (WS)^25^. The WS smoother performed well in multiple cropping regions and therefore was applied here^26^.

### Validation data and other datasets

The validation data in this study included the ground truth reference data and agricultural census data. The ground truth reference data were collected in major agricultural regions with GPS receivers and digital cameras during the study period (2015–2021) (Fig. 1, Table S1). For each sampling site, the geographic location and crop types were recorded. The reliability of ground survey data was improved through visual confirmation based on high-resolution images in Google Earth. Some reference sites with small field sizes were removed to considering the mixed-pixel problems of MODIS images. Finally, we obtained a total of 18,379 ground samples collected during 2015–2021 (Table S1). All the ground truth reference data were used to validate the cropping pattern data in its corresponding year. Agricultural census data were obtained from the National Statistical Bureau of China (NSBC) (http://www.stats.gov.cn/english/), which was collected through sampling statistics. The crop calendar data from agro-meteorological stations recorded the sowing, seedling, tillering, heading, and harvesting dates of winter wheat (210 sites) or spring wheat (90 sites). The calendar data were applied to establish the trend surfaces of key phenological stages of winter wheat and spring wheat, respectively. The crop calendar data were provided by the National Meteorological Information Center, China Meteorological Administration.

The cropland distribution data were derived from the 30 m GlobeLand30 global land cover data in 2020^27^. The total accuracy of GlobeLand30 in 2020 is 85.72%, and the Kappa coefficient is 0.82 (www.globallandcover.com). To correspond to MODIS images, the 30 m cropland pixels from GlobeLand30 data were spatially aggregated to a 500 m cropland fraction map. For simplification, we classified pixel purity of MODIS pixels into three groups: cropland percentages of >90%, 50–90%, and <50% were labeled as pure, moderate mixed, and seriously mixed pixels, respectively. MODIS pixels with very small cropland fraction (i. e. <30%) were not accounted. The pure, moderate mixed and seriously mixed groups occupied 39%, 42%, and 19% of MODIS pixels in China, respectively (Figure S2).

### Overview of the cropping pattern mapping approach

A cropping pattern is referred to as the yearly sequence and spatial distribution of major crops on a specific piece of cropland. Consequently, cropping pattern mapping should provide information on cropping intensity as well as crop types. When multiple cropping is cultivated, we derived the plantation sequence of two or triple crops. For example, the cropping pattern of “winter wheat-maize” represents double cropping with a rotation of winter wheat plus maize. We conducted cropping pattern mapping processes using MATLAB software (Fig. 2). Annual cropping pattern maps were obtained by deriving cropping intensity as well as mapping three staple crop types (paddy rice, maize, wheat). These knowledge-based mapping algorithms were described in the following sections (Fig. 2).

### Deriving cropping intensity

The vegetation indices (VI) peak-based algorithms have been widely applied to identify cropping intensity in previous studies^28^. However, the VI peak-based algorithms were challenged by the changes of VI temporal profiles in different cropping patterns over large areas and multiple years^15,17^. An automatic Cropping Intensity extraction method based on the Isolines of Wavelet Spectra (CIIWS) was proposed with considerations of complex intra-class variability of VI temporal profiles^29,30^. The cropping index was identified based on three main features, the skeleton width, maximum number of strong brightness centers, and the intersection of their scale intervals, derived from wavelet spectra (Fig. 2)^29,30^. The CIIWS cropping intensity mapping algorithm is capable of deriving cropping intensity automatically, which is robust to intra-class variability such as the phenology shift, strengthened or lessened crop growth, or crop diversity^29,30^. The wavelet features-based cropping intensity mapping algorithm was applied in mainland China to derive annual cropping intensity from 1982 to 2013, with an overall accuracy of 91.63%^4^. Therefore, this wavelet features-based cropping intensity mapping algorithm was applied in this study^29,30^.

### Mapping paddy rice, maize and wheat based on phenological indicators

Algorithms for mapping paddy rice, maize and wheat were developed in related references^15,31,32^ and applied in this study. We made critical improvements over these proposed crop mapping algorithms primarily in order to cope with the mixed-pixel problem in MODIS images^15,29–32^. First, pixel purity-based thresholds were applied in the decision rules (Table 1) to cope with the mixed pixel problem of MODIS images. The target crop from mixed pixels was expected to show lower values than those from pure pixels when the target crop was highlighted by larger values in our proposed indicators (i.e., maize). The pixel purity-based thresholds were determined based on the accuracy assessment with agricultural census data in 2018. The pixel purity-based thresholds were applied to a national scale and multiple years (2015–2021) without adaptations. Second, the derived maps were further improved by incorporating their corresponding suitable areas. Specifically, the suitable areas of single rice and spring maize were estimated based on topographic and climatic conditions (elevation and accumulated temperature greater than 10 degrees)^33^. Finally, we calculated the estimated areas of three staple crops from MODIS-derived products using the cropland fraction map. A concise description on the original mapping algorithms were provided in the following sections.

**Table 1:** Information on phenological metrics and decision rules for crop mapping.

Crop	Metrics	Decision rules
Rice	$RCLE=(LSW{I}_{max}-LSW{I}_{min})/(EVI{2}_{heading}-EVI{2}_{tillering})$	$if\;(LSW{I}_{min} > {\theta }_{1}\;and\;RCLE < {\theta }_{2}),\;it\;is\;rice.$
Wheat	${\rm{EVE}}=(EVI{2}_{heading}-EVI{2}_{{\rm{se}}edling})+(EVI{2}_{\max 1}-EVI{2}_{\min 1})$	$if\;\left(EVE > {\theta }_{3}\;and\;{\rm{EVL\; > }}{\theta }_{4}\right),\;it\;is\;winter\;wheat.$
	$${\rm{EVL}}=(EVI{2}_{heading}-EVI{2}_{harvesting}){\rm{+}}(EVI{2}_{\max 2}-EVI{2}_{\min 2})$$	
Maize	$RCPN=({{\rm{P}}}_{early}+{{\rm{P}}}_{late})\times {N}_{late}/({{\rm{N}}}_{early}+{{\rm{N}}}_{late}+\sigma )\times 100$	$if\;(RCPN > {\theta }_{5}),\;it\;is\;maize.$

Notes: RCPN represented the Ratio of Cumulative Positive slope to Negative slope during the flowering stage. RCLE revealed the Ratio of Change amplitude of LSWI to EVI2 from tillering to heading dates. EVE, EVL represented the EVI2 Variations during the Early growth stage and the Late growth stage, respectively. The values of *θ*_2_, *θ*_3_ and *θ*_5_ were {0.42, 0.52, 0.62}, {0.32, 0.24, 0.16}, {0.45, 0.35. 0.25} for pure, moderate, and serious mixed cropland pixels; The values of *θ*_1_ and *θ*_4_ were 0.1 and 0.12, respectively.

A phenology-based rice mapping algorithm was proposed through Combined Consideration of Vegetation phenology and Surface water variations (CCVS)^31^. Variation of LSWI in rice fields was smaller than that in other crops fields during the period from tillering to heading dates^31^. Therefore, the Ratio of Change amplitude of LSWI to 2-band Enhanced Vegetation Index 2 (RCLE) during that period was utilized as the primary metric for paddy rice mapping (Fig. 2, Table 1). The CCVS rice mapping algorithm was successfully applied in 15 provincial-level administrative units of southern China, which obtained an overall accuracy of 93–94%^31^. The CCVS rice mapping algorithm proved to be robust in terms of intra-class rice variability^14^.

An automatic Maize mapping was recently proposed through **E**xploring **L**eaf moisture variation during flowering **S**tage (**MELS**)^32^. One unique indicator for maize mapping was the Ratio of Cumulative Positive slope to Negative slope (*RCPN*) of NMDI during the flowering stage (Fig. 2, Table 1). Maize sites were highlighted by consistently higher values in our proposed phenology-based indicator (*RCPN*) compared to other crops. A simple rule was applied to derive maize^32^ (Table 1). The capability of the MELS method was verified in mainland China, with an overall accuracy of 91%^32^.

A phenology-based winter wheat mapping algorithm through Combining variations Before and After estimated Heading dates (CBAH) (Fig. 2) was exploited in this study^15^. The CBAH algorithm demonstrated the adaptability of VI temporal profiles to intra-class variability^34^. This mapping algorithm was exploited in North China plain from 2001 to 2013, with an overall accuracy of around 90%^15^. The CBAH algorithm was extended to map both winter wheat and spring wheat at the national scale. The potential distribution of winter wheat and spring wheat was determined based on latitudes and the accumulated temperature above 5 °C with references to crop calendar data from agro-meteorological stations. And then the trend surfaces of key phenological stages (heading dates and early growing length) were established for winter wheat and spring wheat, respectively^35^. For winter wheat, altitude and latitude were applied; and for spring wheat, the accumulated temperature above 5 °C and latitude were applied. Two phenology-based indicators were developed by exploring the variations of VI during the estimated early and late growth stages (Fig. 2, Table 1). A simple decision rule could be applied to identify wheat by these two phenology-based indicators (Table 1).

## Data Records

The China Cropping Pattern maps (ChinaCP) are provided during 2015–2021. The datasets are available at the figshare repository in a Geotiff format^35^. The dataset is provided in ESPG: 4326 (WGS_1984) spatial reference system. Classes in the ChinaCP map product are numbered by no more than three digits. The first digit of the classes represents the cropping intensity: {0: fallow; 1: single cropping; 2: double cropping; 3: triple cropping}. The second digit of the single cropping denotes the crop types. The latter two digits of double cropping reveal the rotation of these two specific crop types. The numeric values of these crop types are: {4: maize; 5: paddy rice; 6: wheat; 7: other crops}. Detailed information on the coding of different cropping patterns can be found in Table 2.

**Table 2:** The code and its corresponding cropping patterns in ChinaCP data.

Code	0	14	15	16	17	245	246	255	256	277	3
Cropping pattern	F	SM	SR	SW	SO	R-M	W-M	R-R	W-R	OD	T

Notes: F, SM, SR, SW, SO, D-RM, D-WM, D-RR, D-WR, DO and T represented fallow, single maize, single rice, single wheat, single others, paddy rice-maize, wheat-maize, double rice, wheat-rice, other double-cropping patterns, and triple cropping, respectively.

We also provided the Reference Data for validation (Refer), Crop Calendar data (CCalendar) and the map of Cropland Percentages in each MODIS pixel (CPM) based on GlobeLand30 data in 2020^35^. Crop Calendar data provided the estimated heading dates and early growing length of wheat in China. The maps of Data Availability of ChinaCP (ChinaCP-DA) were revealed by the percentages of valid observations during the growing season (March to October was applied for simplification). Data availability was grouped into three categories: groups 1, 2, and 3, indicating good, medium, and low data availability, which corresponded to >70%, 50–70%, and <50% of valid observations during the growing season, respectively. The datasets of reliability are also provided in Geotiff format (ChinaCP-DA). Mapped results in pixels with low data availability should be applied with caution, especially in southern China (Figure S3).

## Technical Validation

### Site-level comparison with ground-truth data

We compared our maps with ground truth data at each survey site during the period 2015–2021. The overall accuracy (OA), user accuracy (UA), producer accuracy (PA), and F1-score (F1) were calculated for seven annual maps of paddy rice, maize, and wheat using the ground truth data from the survey sites (Table 3). We validated the maps of three staple crops for each year with all the ground survey datasets during its corresponding year. The OA of the three staple crops was 89%. Paddy rice, wheat, and maize were correctly classified with the F1 of 0.91, 0.87, and 0.91, respectively.

**Table 3:** Accuracy assessment using ground reference sites.

MODIS estimates	Reference data						
	Total	Rice	Wheat	Maize	Others	Producer accuracy (%)	F1
Rice	6387	5929	45	56	357	92.83	0.91
Wheat	2657	78	2220	139	220	83.55	0.87
Maize	4662	205	90	4216	151	90.43	0.91
Others	4673	366	85	212	4010	85.81	0.85
User accuracy (%)		90.13	90.98	91.20	84.63		
Overall accuracy (%)	89.10						
Kappa	0.85						

### Province-level comparison with agricultural census data

**Fig. 3:**
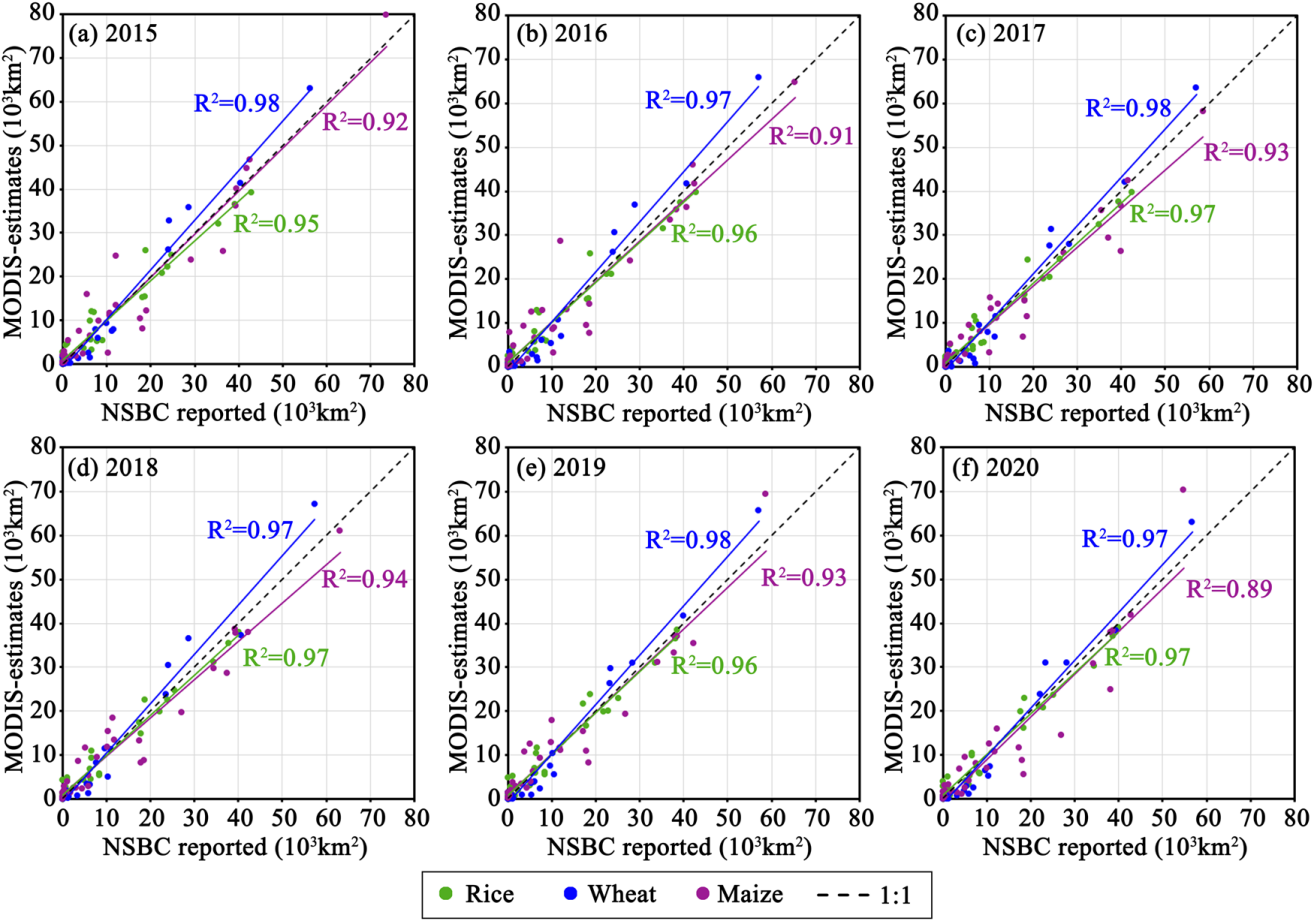
Comparisons between NSBC reports and MODIS-estimates of paddy rice, wheat, and maize from 2015 to 2020 (**a**–**f**).

The sown areas derived from cropping pattern maps were compared with agricultural census data in the yearbook at the provincial level from 2015 to 2020 (Fig. 3). The R^2^ between provincial sown areas of wheat from cropping pattern maps and census data was close to 1 (0.97–0.98) among these six years. The sown areas of paddy rice also agreed well with agricultural census data (R^2^ = 0.95–0.97) (Fig. 3). The coefficients of determination for maize were also no less than 0.89 (0.89–0.94) (Fig. 3). The estimated sown areas of maize in Sichuan or Yunnan provinces showed considerable underestimates compared with the census data (Figure S4, Table S2).

### Sources of error in ChinaCP

Errors and uncertainties in ChinaCP can be attributed to three main groups of sources. The first is the limited data availability due to cloud contamination or other reasons. Data availability illustrated big differences between northern and southern China (Figure S4, Tables S1–S2). The northern portion of China generally obtained much better data availability than that in southern China. Bad data availability during the growing period might introduce commission errors in paddy rice and omission errors in maize (i.e., Sichuan province). The second is associated with the mixed problem of MODIS images. Small-holder farms dominated in China^36^. The mixed-pixel problems might be more serious in mountainous and hilly regions in southern China. Although we proposed several strategies (i.e., pixel-purity-based threshold) to cope with the mixed-pixel problems, there are still uncertainties associated with the seriously mixed pixels.

The third one could be introduced by errors associated with the cropland dataset. The quality of existing cropland products is considerably low due to its high landscape heterogeneity^37^. Additionally, we applied the static cropland mask in 2020 instead of the annually updated cropland during 2015–2021. Cropland in China has experienced tremendous changes such as cropland loss due to urban sprawl and ecological projects (grain for green projects) as well as cropland compensation^38–40^. The mapping accuracy of paddy rice and maize could be influenced by cropland data quality. Specifically, omission errors of cropland could probably introduce underestimation problems, since non-cropland pixels were considered in the classification processes. Commission errors of cropland data would not be associated with overestimation on the condition that the proposed phenology indicators would separate the target crop from other crops as well as non-crop vegetation. Future work could be conducted to propose crop mapping algorithms with no requirements of cropland data, such as the winter wheat mapping algorithm applied in this study^15^.

## Usage Notes

**Fig. 4:**
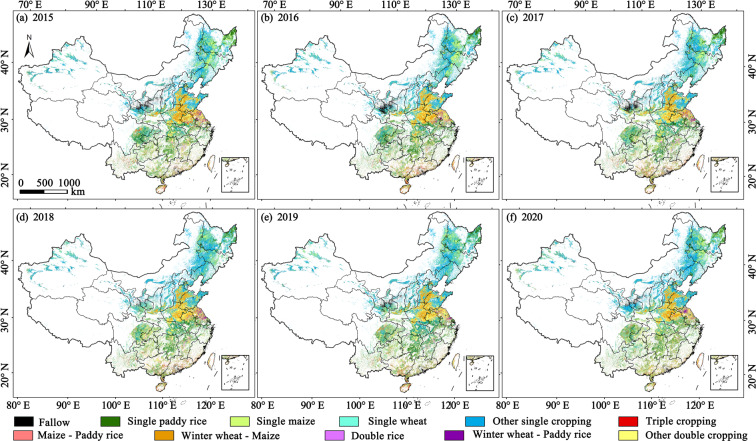
Maps of crop cropping patterns in China from 2015 to 2020 (**a**–**f**).

We provide updated spatiotemporal explicit datasets of major cropping patterns in China during 2015–2021 (Fig. 4). Maps of crop types are critically important for agricultural monitoring systems^41^. National-scale agricultural maps can be applied to assess national food security and promote sustainable agriculture through a better understanding of the impacts from agricultural policies, climatic extreme and agricultural practices, such as the cropping intensity, crop rotations and crop diversification^42,43^. Reliable and updated information on cropping pattern is vital for assessing the changes in cropping patterns under agricultural supply-side reform policy in China. Experiences and lessons from China’s agricultural reform are valuable for the whole world. We recommend users consider the layer of ChinaCP-DA to determine which provinces and years have available high-quality data (Figure S3). Pixels with low data availability should be applied with caution. Year 2020 have much lower data availability compared to other years (Figure S3, Table S3), since the 2020 monsoon has brought historic amounts of rain to China.

## Supplementary information

Supplement

## Data Availability

Time-series images processing and crop mapping algorithms were implemented in MATLAB language. The processing code and related files are available at 10.6084/m9.figshare.14936052. Datasets of cropping patterns in China and other countries/regions with finer resolutions (i.e. 10–30 m) can be produced based on publicly accessible time series images of Landsat and Sentinel-2 Multispectral Instrument (MSI) using the shared processing code^35^.
