# Supplementary material for: Maps of cropping patterns in China during 2015–2021
Source: Sci Data. 2022 Aug 5;9:479. doi: 10.1038/s41597-022-01589-8 (PMC9356131; doi:10.1038/s41597-022-01589-8)
Supplement: Supplementary file 1 — Supplement [file 41597_2022_1589_MOESM1_ESM.docx]

Supplementary Material for:

Qiu, B et al. Maps of cropping patterns in China during 2015-2021. Sci. Data. (2022)

**Table of Contents:**

- Figure S1. Maps of total sown area three staple crops: paddy rice (a), wheat (b), maize (c) and their sums (d) in China in 2020.
- Figure S2. Maps of (a) cropland areas and the percentages of three groups of croplands at provincial level: (b) serious mixed; (c) mixed; (d) pure.
- Figure S3. Maps of percentages of good observation during crop growing season (March to October) from 2015 to 2021 (a-g). and pixels with more than 70% valid observations in 2020 (h).
- Figure S4. Maps of the derivation between MODIS derived results and agricultural census data: (a) paddy rice; (b) wheat; (c) maize during the period 2015-2020.
- Table S1. Information on ground reference sites applied for validation.
- Table S2. Statistical indicators  compared to census data at provincial level.
- Table S3. The percentages of valid observation in cropland in China from 2015 to 2021.


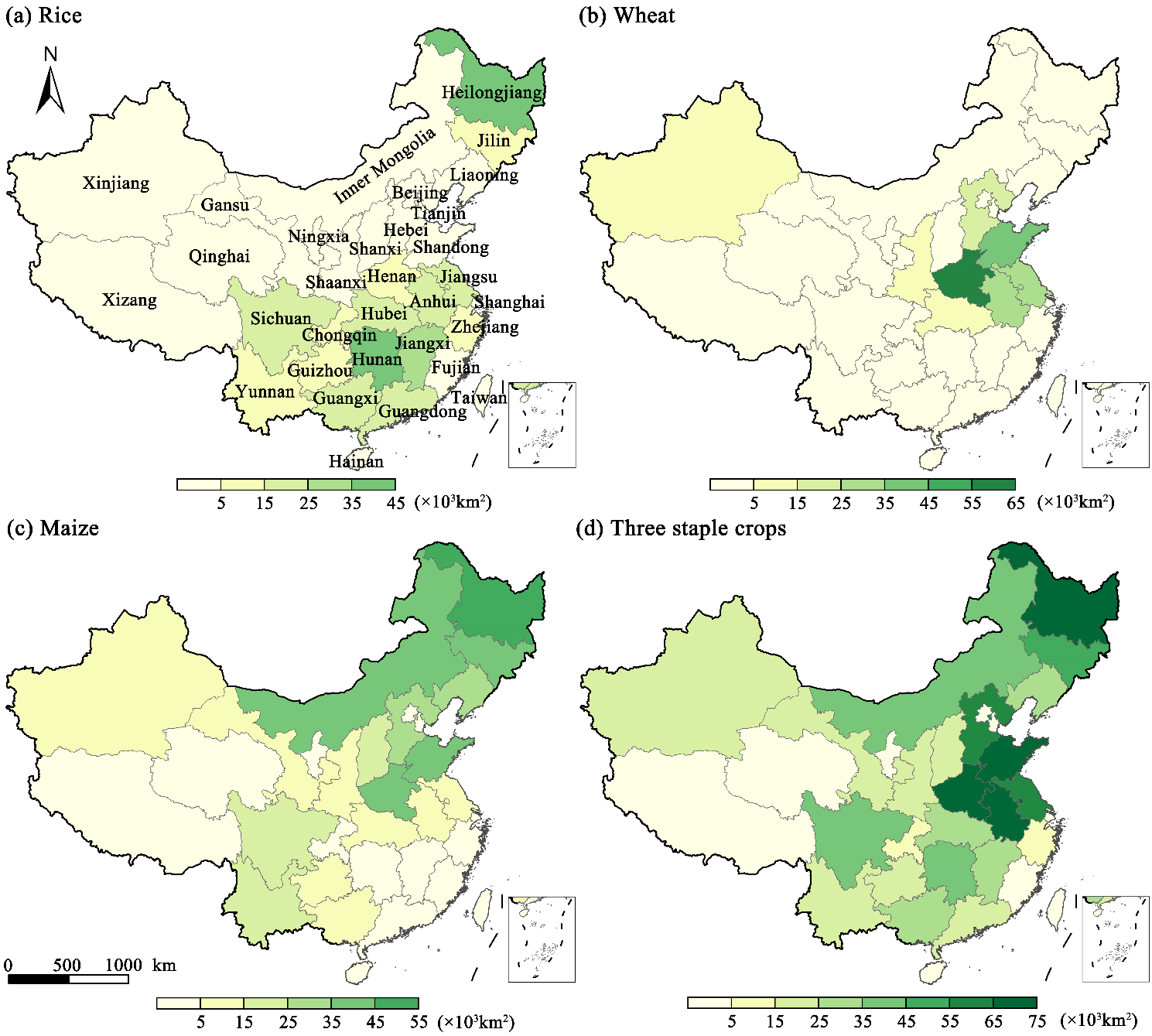


###### Figure S1. Maps of total sown area three staple crops: paddy rice (a), wheat (b), maize (c) and their sums (d) in China in 2020


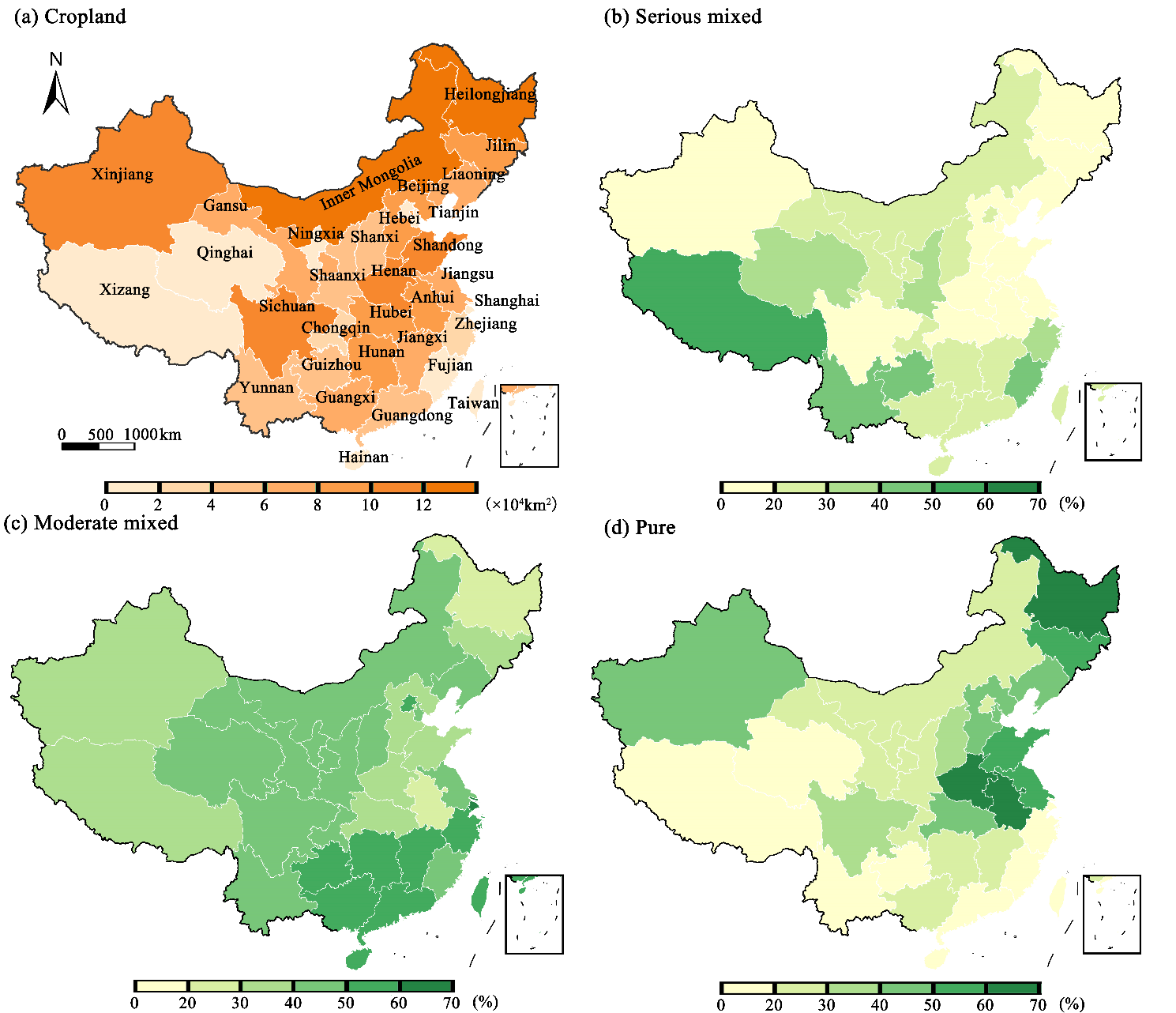


###### Figure S2. Maps of (a) cropland areas and the percentages of three groups of croplands at provincial level: (b) serious mixed; (c) mixed; (d) pure.


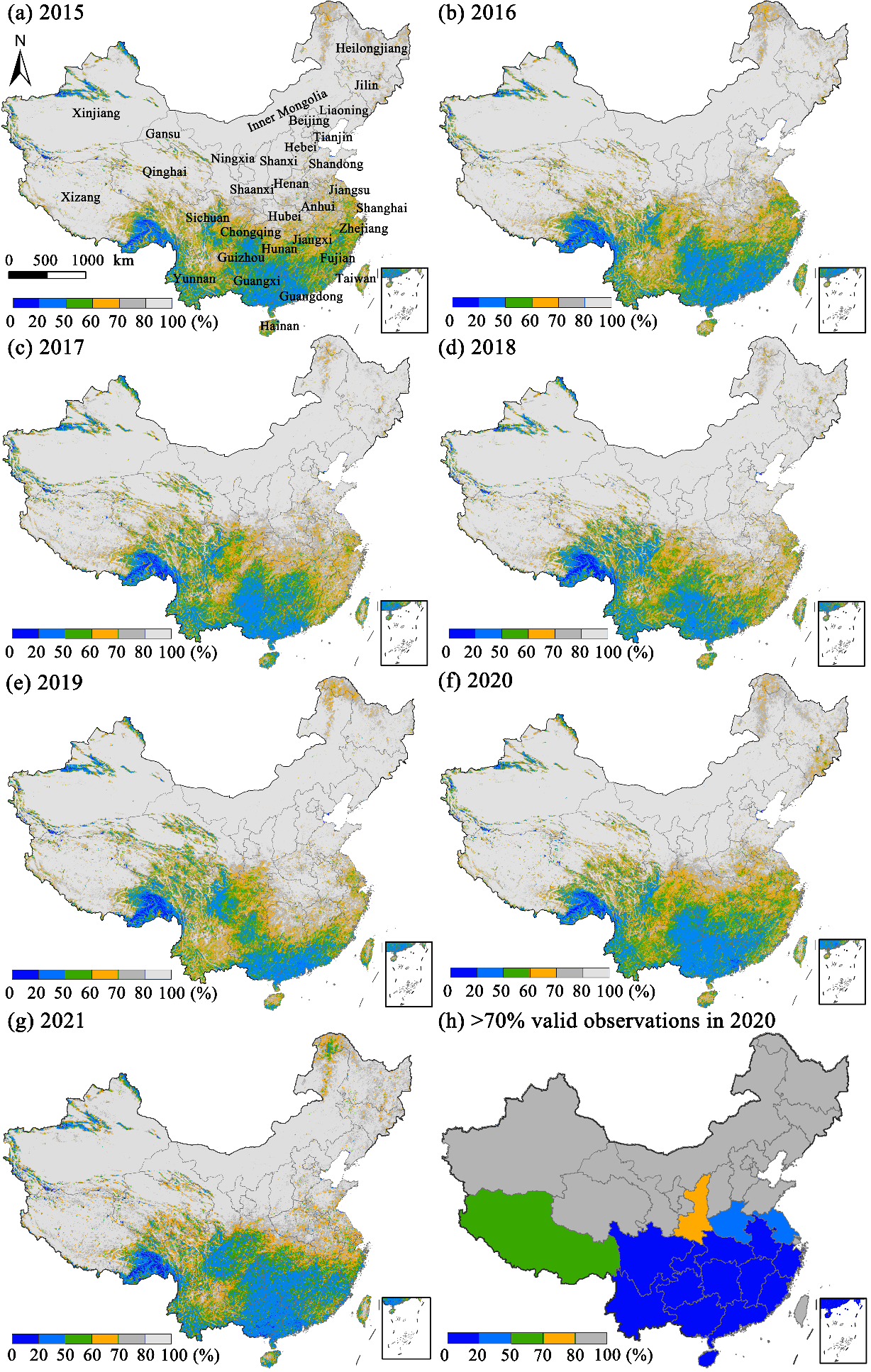


###### Figure S3. Maps of percentages of good observation during crop growing season (March to October) from 2015 to 2021 (a-g). and pixels with more than 70% valid observations in 2020 (h).


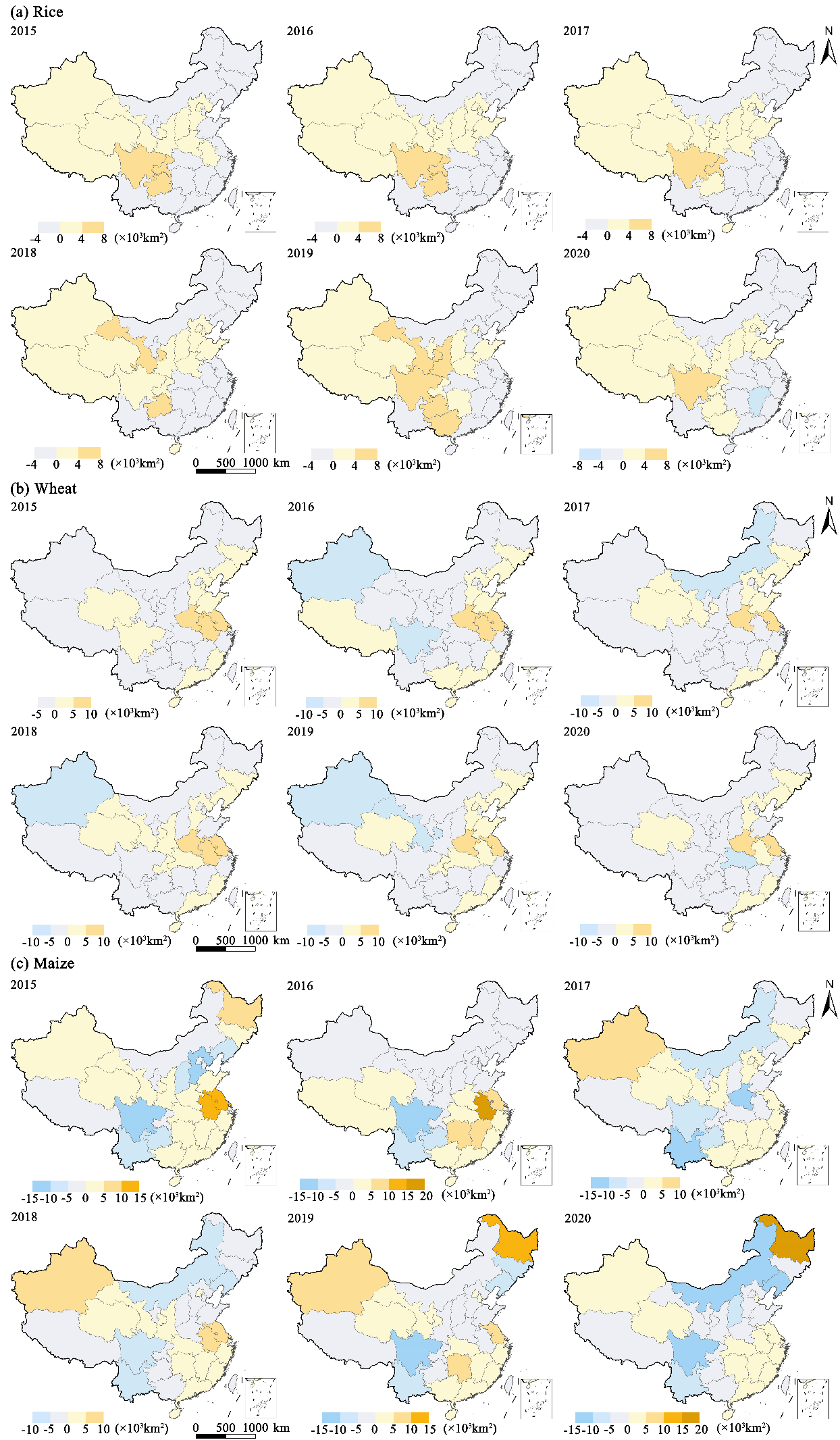


###### Figure S4. Maps of the derivation between MODIS derived results and agricultural census data: (a) paddy rice; (b) wheat; (c) maize during the period 2015-2020.

###### Table S1. Information on ground reference sites applied for validation

|  | 2015 | 2016 | 2017 | 2018 | 2019 | 2020 | 2021 | Total |
| --- | --- | --- | --- | --- | --- | --- | --- | --- |
| Rice | 1023 | 602 | 871 | 935 | 728 | 769 | 1650 | 6578 |
| Wheat | 323 | 230 | 294 | 310 | 281 | 288 | 714 | 2440 |
| Maize | 756 | 499 | 467 | 644 | 601 | 675 | 981 | 4623 |
| Other | 730 | 503 | 356 | 465 | 455 | 434 | 1795 | 4738 |
| Total | 2832 | 1834 | 1988 | 2354 | 2065 | 2166 | 5140 | 18379 |

###### Table S2. Statistical indicators  compared to census data at provincial level

| Crop | Year | Deviation (km^2^) | | | | RMSE | Deviation rate | R^2^ |
| --- | --- | --- | --- | --- | --- | --- | --- | --- |
|  |  | Mean | Median | Maximum | Minimum |  |  |  |
| Rice | 2015 | -37 | -231 | 7087 | -3776 | 2671 | -0.38% | 0.95 |
|  | 2016 | 66 | -494 | 7045 | -3736 | 2526 | 0.67% | 0.96 |
|  | 2017 | -119 | -178 | 5592 | -3345 | 2275 | -1.20% | 0.97 |
|  | 2018 | -88 | -88 | 4213 | -3276 | 2154 | -0.90% | 0.97 |
|  | 2019 | 266 | -131 | 5007 | -2891 | 2315 | 2.78% | 0.96 |
|  | 2020 | 70 | -503 | 4174 | -4124 | 2104 | 0.72% | 0.97 |
| Wheat | 2015 | 78 | -112 | 8627 | -4766 | 2769 | 1.00% | 0.98 |
|  | 2016 | -94 | -237 | 8852 | -5509 | 3065 | -1.00% | 0.97 |
|  | 2017 | -131 | -79 | 7188 | -6145 | 2571 | -2.00% | 0.98 |
|  | 2018 | 57 | -43 | 9722 | -5328 | 2907 | 1.00% | 0.97 |
|  | 2019 | -88 | -17 | 8677 | -5180 | 2611 | -1.00% | 0.98 |
|  | 2020 | -490 | -179 | 7618 | -5131 | 2714 | -6.00% | 0.97 |
| Maize | 2015 | 30 | 313 | 12675 | -10866 | 4885 | 0.23% | 0.92 |
|  | 2016 | -53 | -153 | 16524 | -11085 | 4862 | -0.41% | 0.91 |
|  | 2017 | -802 | 63 | 5471 | -13672 | 4168 | -6.43% | 0.93 |
|  | 2018 | -649 | 0 | 7056 | -9771 | 4110 | -5.24% | 0.94 |
|  | 2019 | 5 | 153 | 10812 | -10221 | 4205 | 0.04% | 0.93 |
|  | 2020 | -1050 | -2 | 15599 | -13294 | 5352 | -8.65% | 0.89 |

###### Table S3. The percentages of valid observation in cropland in China from 2015 to 2021

| Year | Percentages of valid observations | |
| --- | --- | --- |
|  | <70 | >70 |
| 2015 | 32.02% | 67.98% |
| 2016 | 34.44% | 65.56% |
| 2017 | 32.86% | 67.14% |
| 2018 | 28.95% | 71.05% |
| 2019 | 27.32% | 72.68% |
| 2020 | 39.94% | 60.06% |
| 2021 | 33.55% | 66.45% |
